# Early immune adaptation in HIV-1 revealed by population-level approaches

**DOI:** 10.1186/s12977-014-0064-1

**Published:** 2014-08-29

**Authors:** Eric Martin, Jonathan M Carlson, Anh Q Le, Denis R Chopera, Rachel McGovern, Manal A Rahman, Carmond Ng, Heiko Jessen, Anthony D Kelleher, Martin Markowitz, Todd M Allen, M-J Milloy, Mary Carrington, Mark A Wainberg, Zabrina L Brumme

**Affiliations:** Faculty of Health Sciences, Simon Fraser University, Burnaby, BC Canada; British Columbia Centre for Excellence in HIV/AIDS, Vancouver, BC Canada; Microsoft Research, Los Angeles, CA USA; KwaZulu-Natal Research Institute for Tuberculosis and HIV, Nelson R. Mandela School of Medicine, University of KwaZulu-Natal, Durban, South Africa; Jessen-Praxis, Berlin, Germany; Kirby Institute, University of New South Wales, Sydney, Australia; Aaron Diamond AIDS Research Center, The Rockefeller University, New York, NY USA; Ragon Institute of MGH, MIT and Harvard University, Cambridge, MA USA; Faculty of Medicine, University of British Columbia, Vancouver, BC Canada; Cancer and Inflammation Program, Laboratory of Experimental Immunology, Leidos Biomedical Research Inc, Frederick National Laboratory for Cancer Research, Frederick, MD USA; Lady Davis Institute, McGill University, Montreal, Canada

**Keywords:** Human immunodeficiency virus type-1 (HIV-1), Human leukocyte antigen (HLA) class I, CD8+ cytotoxic T-lymphocytes (CTL), Immune escape, HLA-associated polymorphism, Adaptation, Evolution, Acute/early infection, Population-level analysis, Statistical association with phylogenetic correction

## Abstract

**Background:**

The reproducible nature of HIV-1 escape from HLA-restricted CD8+ T-cell responses allows the identification of HLA-associated viral polymorphisms “at the population level” – that is, via analysis of cross-sectional, linked HLA/HIV-1 genotypes by statistical association. However, elucidating their timing of selection traditionally requires detailed longitudinal studies, which are challenging to undertake on a large scale. We investigate whether the extent and relative timecourse of immune-driven HIV adaptation can be inferred via comparative cross-sectional analysis of independent early and chronic infection cohorts.

**Results:**

Similarly-powered datasets of linked HLA/HIV-1 genotypes from individuals with early (median < 3 months) and chronic untreated HIV-1 subtype B infection, matched for size (N > 200/dataset), HLA class I and HIV-1 Gag/Pol/Nef diversity, were established. These datasets were first used to define a list of 162 known HLA-associated polymorphisms detectable at the population level in cohorts of the present size and host/viral genetic composition. Of these 162 known HLA-associated polymorphisms, 15% (occurring at 14 Gag, Pol and Nef codons) were already detectable via statistical association in the early infection dataset at p ≤ 0.01 (q < 0.2) – identifying them as the most *consistently* rapidly escaping sites in HIV-1. Among these were known rapidly-escaping sites (*e.g.* B*57-Gag-T242N) and others not previously appreciated to be reproducibly rapidly selected (*e.g.* A*31:01-associated adaptations at Gag codons 397, 401 and 403). Escape prevalence in early infection correlated strongly with first-year escape rates (Pearson’s R = 0.68, p = 0.0001), supporting cross-sectional parameters as reliable indicators of longitudinally-derived measures. Comparative analysis of early and chronic datasets revealed that, on average, the prevalence of HLA-associated polymorphisms more than doubles between these two infection stages in persons harboring the relevant HLA (p < 0.0001, consistent with frequent and reproducible escape), but remains relatively stable in persons lacking the HLA (p = 0.15, consistent with slow reversion). Published HLA-specific Hazard Ratios for progression to AIDS correlated positively with average escape prevalence in early infection (Pearson’s R = 0.53, p = 0.028), consistent with high early within-host HIV-1 adaptation (via rapid escape and/or frequent polymorphism transmission) as a correlate of progression.

**Conclusion:**

Cross-sectional host/viral genotype datasets represent an underutilized resource to identify reproducible early pathways of HIV-1 adaptation and identify correlates of protective immunity.

**Electronic supplementary material:**

The online version of this article (doi:10.1186/s12977-014-0064-1) contains supplementary material, which is available to authorized users.

## Background

HIV-1 escape from Human Leukocyte-Antigen (HLA) class I-restricted CD8+ T-lymphocytes (CTL) occurs in a broadly predictable manner based on the HLA alleles expressed by the host [1]. Reversion of escape mutations, usually to consensus, upon HIV-1 transmission to an individual lacking the restricting HLA also occurs reproducibly in many [2-5], though not all [6-8], cases. The reproducible nature of viral adaptation allows us to identify HLA-associated polymorphisms in HIV-1 (that is, viral polymorphisms that are significantly over- or under- represented among persons expressing a given HLA allele) “at the population level” (that is, via the analysis of cross-sectional, linked HLA/HIV-1 genotypes via statistical association approaches that additionally correct for various potential confounders [9-12]). Such studies are normally undertaken in chronic infection, as the virus has undergone a majority of its within-host adaptation by this stage. For example, a recent population-level study of >1800 chronically HIV-1 subtype B-infected persons identified over >2000 HLA-associated polymorphisms across HIV-1, with a majority occurring in Gag, Pol and Nef [11].

Though HLA-associated polymorphisms in HIV-1 can be identified using cross-sectional approaches, their timing of selection cannot be directly determined by these methods*.* Rather, temporal information is ideally established via detailed longitudinal study of untreated individuals recently infected with HIV-1 (*e.g.*: [2,3,13-19]). However, identifying large numbers of recently-infected persons is challenging. Another consideration is that, given the current evidence and clinical recommendations supporting HIV-1 treatment initiation in early infection [20], prospective longitudinal observational study of untreated HIV-1 infection may no longer be feasible nor ethical moving forward.

As such, cross-sectional pretreatment host/viral genotype datasets from individuals at different HIV-1 infection stages enrolled in established (or future) cohorts could potentially provide alternate data sources to infer the extent and time course of immune-driven HIV-1 adaptation, including the earliest events post-infection, using population-level approaches. Though such approaches have been investigated [21,22], they remain underutilized in this context. Notably, population-level approaches offer one key advantage in that - by definition - they specifically identify HIV-1 adaptations that occur *reproducibly* in persons expressing the restricting HLA [21] (as opposed to longitudinal studies that characterize immune escape dynamics in individual persons, but cannot elucidate the extent to which such pathways are shared between persons, *e.g.* [3,17-19]). As such, population-level studies may be particularly useful in identifying the HLA-restricted CTL escape mutations that are most rapidly *and reproducibly* selected following HIV-1 infection.

In an attempt to achieve these goals, we undertook a proof-of-concept study that compared the prevalence of known HLA-associated polymorphisms in HIV-1 Gag, Pol and Nef [11] in identically-sized cross-sectional early and chronic infection cohorts that were matched as closely as possible for their HLA allele distributions and their total HIV-1 diversity. Our main goals were: 1) to assess the utility of population-level approaches to identify the most reproducibly rapid escape mutations in HIV-1; 2) to estimate the extent of escape and reversion between early and chronic infection; and 3) to investigate whether features related to population-level early immune escape signal can discriminate protective from non-protective HLA alleles.

## Results and discussion

### Assembling early and chronic infection cohorts matched for size, HLA and HIV-1 diversity

Our study sought to demonstrate that the extent, reproducibility and relative timing (early versus later) of HLA-driven escape in HIV-1 can be inferred via comparative analysis of independent cross-sectional host/virus genotype datasets from different infection stages. This strategy ideally requires cross-sectional datasets that are identically powered with respect to host and viral genetic diversity (*i.e.* datasets that mimic longitudinal data as closely as possible, in that they differ only with respect to infection stage of the participants). As such, our first step was to assemble early and chronic HIV-1 subtype B cohorts of identical size that were matched as closely as possible for HLA class I allele distribution and HIV-1 diversity. We did so by drawing upon host and viral genotype data from early and chronic infection cohorts in North America, Europe and Australia (methods and [13,23-25]). Our final early and chronic datasets comprised 221 Gag, 203 Pol and 219 Nef HIV-1 subtype B sequences *per cohort*, for which linked HLA class I types were available. Early cohort patients were recruited a median of 88 [IQR 63–120] days following infection. All early and >75% of chronic patients were antiretroviral naïve; the remainder were untreated at time of sampling.

**Figure 1:**
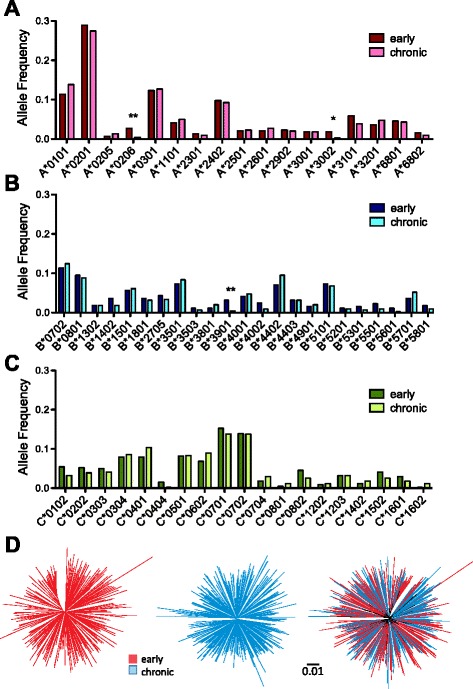
**Early and chronic datasets are comparable with respect to host and viral diversity.** The 17 HLA-A, 23 HLA-B and 19 HLA-C alleles (total 56) observed at frequencies > 1% in the early and/or chronic datasets are displayed in ***Panels A***-***C***, respectively. The early and chronic datasets were comparable with respect to all HLA allele frequencies except HLA-A*02:06, A*30:02 and B*39:01 whose frequencies were higher in the early cohort compared to the chronic cohort (denoted by “*” for p < 0.05 and “**” for p < 0.01, Fisher’s exact test). Note however that no HIV-1 polymorphisms restricted by these three HLA class I alleles were assessed in the present study (see Figure 2 and Additional file 1). ***Panel D***: Unrooted maximum-likelihood phylogenies of early (left), chronic (middle) and combined cohort (right) Gag sequences, on a distance scale of 0.01 substitutions per nucleotide site. Mean patristic (pairwise) genetic distances between Gag sequences were comparable for early and chronic cohorts; moreover, no gross cohort-specific clustering is observed in the combined phylogeny.

A total of 59 HLA class I alleles, classified at subtype-level (4-digit) resolution, were observed at a frequency >1% in the early and/or chronic cohorts; these comprised 17 HLA-A, 23 HLA-B and 19 HLA-C alleles (Figure 1). Of these 59 alleles, the frequencies of 56 (94.9%) were comparable between cohorts; only three alleles (HLA-A*02:06, A*30:02 and B*39:01) exhibited significantly different frequencies between cohorts (all p < 0.05; 0.08 < q < 0.33) (Figure 1A-C). As such, our early and chronic cohorts were generally well-matched with respect to host HLA diversity.

HIV-1 Gag, Pol and Nef diversity was also generally comparable between the two cohorts. Mean patristic (pairwise) genetic distances between HIV-1 sequences in early versus chronic datasets, measured in units of substitutions per nucleotide site, were 0.076 (Standard Deviation [SD] ± 0.011) versus 0.071 (SD ± 0.010) respectively for Gag (Figure 1D left and middle panels), 0.057 (SD ± 0.008) versus 0.053 (SD ± 0.008) for Pol, and 0.119 (SD ± 0.018) versus 0.120 (SD ± 0.021) for Nef (not shown). Moreover, no gross inter-cohort segregation was observed in a combined HIV-1 Gag phylogeny (Figure 1D, right), indicating that neither cohort was dominated by large epidemiologically linked clusters nor exhibited evidence of recent descent from distinct ancestors. Together, these data suggest that our early and chronic datasets are similarly powered with respect to host and viral genetic diversity, and thus differ only with respect to infection stage.

### Defining the list of HLA-associated polymorphisms for investigation in cohorts of the present size and composition

A total of 453 HLA-associated polymorphisms in Gag/Pol/Nef had previously been identified at q < 0.05 in an independent cohort of N > 1800 individuals with chronic HIV-1 subtype B infection [11], which contained no overlap with the cohorts studied here. These HLA-associated polymorphisms comprise “adapted” associations (HIV-1 amino acids significantly *over-represented* in the presence of the HLA allele in question) as well as “nonadapted” associations (HIV-1 amino acids significantly *under-represented* in the presence of the HLA allele). For example, at Gag codon 242 the nonadapted amino acid associated with HLA-B*57:01 is the subtype B consensus “T” whereas the B*57:01 adapted form is “N”, denoted as “B*57:01-Gag-T242N”. The cohort wherein these HLA-associations were originally defined however [11] was more than seven times larger than the cohorts presently studied. Therefore, we do not have sufficient statistical power to interrogate all of them in the present study. As such, our next step was to define, from the published list [11], the subset of known HLA-associated polymorphisms that is appropriate for study in cohorts of the present size and host/viral genetic composition.

**Figure 2:**
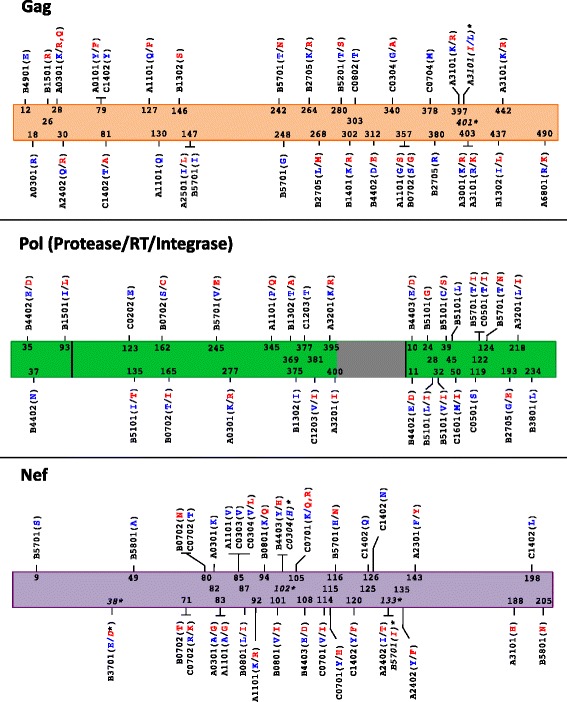
**HLA-associated polymorphisms detectable at the population level in cohorts of the present size and genetic composition.** Gag, Pol and Nef “immune escape maps” indicate the codon location, specific amino acid residues and HLA restrictions of the N = 162 known HLA-associated polymorphisms detectable at the population level in cohorts of the present size and host/viral genetic composition. “Nonadapted” amino acids (those *under-represented* in the presence of the HLA allele) are blue; these represent the “immunologically susceptible” form for the HLA allele in question (and usually represent the subtype consensus residue). “Adapted” amino acids (those *over-represented* in the presence of the HLA allele) are red; these represent the HLA-associated “escape variant”. Adapted and Nonadapted associations are counted independently; in most cases both forms are detectable at the population level at a given p-value threshold (*e.g.* at Gag codon 242, “T” and “N” represent the B*57:01 – associated nonadapted and adapted forms, respectively), whereas in other cases only one of the two forms is detectable at a given threshold (*e.g.* at Gag codon 12, “E” represents the B*49:01-associated nonadapted form but no specific adapted form is detected at this threshold). Asterisks (*) and *italicized text* denote the five HLA-associations at Gag codons 401 and Nef codons 38, 102 and 133 that were defined via detection in the early cohort at p < 0.01, but were p ≥ 0.01 in the chronic cohort. Note that HIV-1 RT genotyping was performed for codons 1–400 of this protein only (the remainder of RT is colored gray). Subsequent analyses focused on this list of HLA-associated polymorphisms.

Theoretically, if all immune escape mutations, once selected, persisted for the remainder of the host’s lifetime, and if we had achieved perfect genetic matching between our early and chronic cohorts, then we could define an appropriate subset of HLA-associated polymorphisms by interrogating our chronic cohort for the presence of these N = 453 known HLA-associated polymorphisms. Those detectable at the population level in chronic infection, a stage when a majority of within-host adaptation has already occurred, would represent an appropriate subset for study in cohorts of the present size. Thus we first interrogated our chronic cohort for the presence of these 453 published HLA-associated polymorphisms using statistical association with phylogenetic correction (see Methods and [12,26]), and in doing so identified 157 (35%) “adapted” and “nonadapted” HLA associations at p < 0.01 (corresponding to q < 0.01 in this analysis) (Figure 2 and Additional file 1). These comprised 54, 52 and 51 HLA-associated polymorphisms in Gag, Pol and Nef respectively.

However, if genetic matching of early and chronic cohorts was imperfect, and/or if immune escape mutations occasionally arose transiently during infection, then defining HLA-associated polymorphisms based on the chronic cohort only could potentially lead us to miss some associations. Thus, we interrogated our early cohort for this same list of published HLA-associated polymorphisms using identical methods. In doing so we identified an additional 5 HLA-associated polymorphisms at p < 0.01, two occurring at Gag codon 401 and one each occurring at Nef codons 38, 102 and 133, that had not been identified in the chronic analysis (Figure 2 and Additional file 1).

We therefore took the union of these results, totaling N = 162 HLA-associated polymorphisms, as our definition of known HLA-associated polymorphisms appropriate for cohorts of the present size and composition (Figure 2 and Additional file 1). To further validate this subset, we applied a published phylogenetically-corrected interaction test (see Methods and [12,27]) to compare the strengths of selection of these individual HLA-associated polymorphisms in early versus chronic cohorts. Given that within-host HIV-1 adaptation increases over the infection course via the selection of immune escape mutations, we would expect higher strengths of association between the restricting HLA and the HIV-1 polymorphism at later versus earlier stages: indeed, of the 162 HLA-associated polymorphisms studied, the strengths of 101 (62%) of them differed significantly (p < 0.01, q ≤ 0.1) between early and chronic infection (Additional file 1). Note also that none of the N = 162 polymorphisms were restricted by the three HLA alleles (A*02:06, A*30:02 and B*39:01) whose frequencies differed significantly between cohorts. All downstream analyses therefore focused on this list of N = 162 HLA-associated polymorphisms.

### Fifteen percent of known HLA-associated polymorphisms are already detectable at the population level in early infection

Our first objective was to assess the extent of population-level signal for HLA-driven escape in early HIV-1 infection. Of the N = 162 HLA-associated polymorphisms identified for study, 24 (15%), occurring at 14 unique codons in Gag, Pol and Nef, were detectable at the population level in the early cohort at a threshold of p ≤ 0.01 (Table 1). In total, these 24 associations comprised 16% of those investigated in Gag (9 of 56), 6% of those investigated in Pol (3 of 52) and 22% of those investigated in Nef (12 of 54).

**Table 1:** **HLA-associated polymorphisms detectable at the population level in early HIV-1 infection**

**Protein**	**HLA**	**HIV polymorphism** ^***a***^	**Early infection**		**Chronic infection**		**CD8+ epitope** ^*^	**p-value (early vs. chronic)** ^***d***^
			**Odds Ratio** ^***b***^	**p-value** ^***c***^	**Odds Ratio** ^***b***^	**p-value** ^***c***^	**(HIV codon coordinates)**	
Gag	B*57:01	**T242N**	33	3 × 10^−9^	151	6 × 10^−17^	TS***T***LQEQIGW (240–248)	0.3
	C*07:04	**M378x**	3.6	0.006	3.5	0.0002	I***M***MQRGNF (377–383)*	0.7
	A*31:01	**K397R**	6.2	1 × 10^−7^	83	8 × 10^−10^	CG***K***EGHIAR (395–403)	0.5
	A*31:01	*I401L*	5.1	0.005	1.98	0.01	CGKEGH***I***AR (395–403)	0.2
	A*31:01	**R403K**	1.5	0.007	~41	1 × 10^−7^	CGKEGHIA***R*** (395–403)	0.002
Pol (RT)	B*51:01	**I135x**	2.1	0.004	25	5 × 10^−11^	TAFTIPS***I*** (128–135)	0.0001
Pol (Int)	B*51:01	**L28I**	16	0.009	5	7 × 10^−5^	***L***PPIVAKEI (28–36)	0.2
Nef	B*37:01	**E38** *D*	13	0.006	~24	0.002	L***E***KHGAIT (37–45)*	0.2
	C*03:04	**V85L**	2.4	0.002	4	0.0002	AA***L***DLSHFL (83–91)	0.6
	A*11:01	**K92R**	5.3	0.009	8.4	4 × 10^−6^	AVDLSHFL***K*** (84–92)	0.2
	C*03:04	*H102x*	3.2	0.001	~1	0.25	none	0.1
	A*23:01	**F143Y**	8.4	0.01	852	5 × 10^−8^	RYPLTFGWC***F*** (134–143)	0.1
	B*57:01	*x133I*	6	0.004	1.4	0.29	YTPGPG***I***RY (127–135)	0.2
	A*24:02	**Y135F**	2.3	0.004	15	2 × 10^−21^	R***Y***PLTFGW (134–141)	0.0005

^*a*^A total of 24 associations, occurring at 14 unique HIV-1 codons, are listed. The total 24 is reached because HLA-associated nonadapted and adapted forms are counted individually (*e.g.* B*57:01-Gag-T242N comprises two associations– the nonadapted T and the adapted N). Cases where a specific non-adapted or adapted form was not detected in early infection are denoted by a lowercase “*x*” (*e.g.* B*51:01-RT-I135*x*). Polymorphisms in **bold** represent associations detectable in both early and chronic infection at p ≤ 0.01; those *italicized* represent associations detectable in only the early cohort with p < 0.01.

^*b*^Where both nonadapted and adapted forms for a given HLA are identified, the maximum absolute Odds Ratio is shown.

^*c*^Where both nonadapted and adapted forms for a given HLA are identified, the lowest p-value is shown. Note the chronic p-value for B*37:01-Nef-E38D refers to its nonadapted (E38x) form; the p-value for the adapted (x38*D*) form at this stage is 0.07.

^*^Bioinformatically predicted CTL epitopes are denoted by asterisks (*); the remainder are published (http://www.hiv.lanl.gov/content/immunology/tables/tables.html). Bold letters indicate the position within the epitope where the HLA-associated polymorphism occurs. Note the C*03-restricted AALDLSHFL epitope has been published in its C*03-adapted form.

^*d*^For each HLA-associated polymorphism in the table, its strength association in early versus chronic infection was compared using a previously-described phylogenetically-corrected interaction test (see Methods and [12,27]). The p-values of these comparisons are listed in this column.

As expected, among these were escape mutations known or previously observed to occur in the first year of infection, including B*57:01 Gag-T242N, B*51:01 RT-I135X and Int-L28I, C*03:04 Nef-V85L, A*11:01 Nef-K92R and A*24:02 Nef-Y135F [2,13,16,18,19,21,28]. Our findings therefore provide proof-of-concept that the most consistently rapid host adaptations in HIV-1 can be identified using cross-sectional methods. It is notable that, by < 3 months post-infection, the magnitude of statistical association between certain HLA alleles and their associated viral polymorphisms is already very high, and in some cases not significantly different from their magnitudes of association in chronic infection. For example, the Odds Ratio [OR] of association between B*57 and Gag-T242N is 33 in early infection (p = 3 × 10^−9^) compared to 151 (p = 5 × 10^−16^) in chronic infection, which, though stronger during the latter stage, does not represent a statistically significant difference (inter-cohort comparison p = 0.3) (Table 1). This observation underscores the rapid and highly reproducible nature of certain HLA-driven within-host adaptations in HIV-1, where, for certain mutations such as Gag-T242N, escape (and by extension our ability to detect this association via population-level methods) is already near maximal in B*57:01-expressing persons < 3 months post-infection.

Of note, HLA-associated HIV-1 polymorphisms with strong early population-level escape signal also included understudied viral sites. Notable among these were A*31:01-associated polymorphisms at Gag codons 397, 401 and 403, the first of which represented the second strongest p-value detected in the early cohort (Odds Ratio = 6.2, p = 1 × 10^−7^, Table 1). These associations are located within the novel A*31:01-restricted CR9 CD8+ epitope originally characterized via detailed longitudinal analysis of a single HIV-1 subtype B-infected individual [18,19]. By definition, population-level studies identify viral adaptations that occur reproducibly in persons expressing the restricting HLA; as such, the present results extend those of the original individual-level study [18] by indicating that escape within CR9 is both rapid and highly consistent in HLA-A*31:01-expressing persons. By extension, a lack of population-level early escape signal does not necessarily mean that a given site never escapes early: rather, it indicates that a given site does not *reproducibly* escape early (or at least does not do so to an extent that achieves statistical significance in a dataset of the present size). For example, very rapid (<30 days) escape was previously documented within the A*01-restricted GY9 epitope (Gag codons 71–79) in two HIV-1 subtype C-infected persons [19], but no evidence of reproducible early escape Gag codon 79 in A*01-expressing persons was observed in our early dataset, suggesting that rapid A*01-driven escape at this position is atypical in HIV-1 subtype B.

Taken together, population-level analyses extend those of individual-level studies by identifying escape mutations that are rapidly *and reproducibly* selected across patients. Our observation that 15% of known HLA-associated polymorphisms, notably those in Nef and Gag, are already detectable < 3 months post-infection, underscores the predictable and rapid nature of HIV-1 adaptation despite each individual’s unique combination of host HLA and transmitted virus genetics. Further, the detection of substantial population-level escape signal within unknown or understudied CD8+ epitopes in HIV-1 (Table 1) argues for continued efforts to map novel epitopes commonly targeted during this critical infection stage.

### Can population-level approaches identify transient early escape pathways?

Recent longitudinal studies have revealed that immune escape is often characterized by the initial appearance of transient mutant forms that often retain some ability to be targeted by existing (or *de novo*) CTL [18,29], which then drive the selection of more effective escape variants that ultimately become fixed within the host [3,17,18]. If such “transient” escape pathways are reproducible across hosts, we wondered whether population-level approaches could theoretically be used as exploratory tools to identify them. If so, we reasoned that such transient escapes would display *stronger* population-level escape signal in early compared to chronic infection (since, in some persons, the early variant would be subsequently replaced with another, thereby reducing population-level signal in later stages). Although our inter-cohort comparative analysis revealed no HLA-associated polymorphisms that displayed significantly stronger signal in early versus chronic infection (Additional file 1 and data not shown), we were nevertheless intrigued by the five HLA-associated polymorphisms in Gag and Nef that exhibited population-level escape signal of p < 0.01 in our early cohort but p ≥ 0.01 in the chronic cohort (Table 1), suggesting these as possible transient escape pathways.

Indeed, analysis of available longitudinal bulk plasma HIV-1 RNA Nef sequences from seven B*57:01 expressing individuals identified one case where an individual harbored the Nef-133I adapted mutation at the earliest sampled timepoint 30 days post-infection, which was replaced by V at 86 days post-infection and then by a mixture of I/V at 228 days-post infection (Table 2). Similarly, analysis of available bulk plasma Gag sequences from seven A*30:01 expressing persons identified one case where an individual harbored the adapted Gag-401 L variant at the earliest timepoint post-infection, that was subsequently replaced by a non-adapted form (and/or a mixture of the two) within a year of infection. In both of these cases the association is located at position 7 within the epitope, which is consistent with transient early escape mutations representing incomplete TCR repertoire escape variants [18,29]. The idea that escape mutations, once selected, may not always persist for the lifetime of the host is also supported by within-host reversion of certain escape mutations in very advanced disease [22]. We thus cautiously interpret the data to suggest that cross-sectional approaches could theoretically be used to identify reproducible HLA-driven adaptations that represent “transient” early escape variants in some individuals, though such findings would require validation in independent cohorts, as well as experimentally.

**Table 2:** **Examples of possible transient early HLA-driven escape at HIV codons with stronger population-level signal in early versus chronic infection**

**HLA-associated HIV-1 polymorphism**	**Patient HLA**	**Days post-infection**	**Bulk plasma seq.**	**Adapted to HLA?**
A*31:01-Gag-I401L	A0301/***3101*** B4403/3503 C0401/0401	143	***L***	yes
		226	***L***	yes
		309	***I***	no
		485	***I/L***	partial
		563	***I***	no
		683	***I/L***	partial
B*57:01-Nef-*x*133I	A2402/2902 B4403/***5701*** C0602/1601	30	***I***	yes
		31	***I***	yes
		60	***I***	yes
		86	***V***	no
		123	***V***	no
		228	***[I/V]***	partial
		361	***[I/V]***	partial
		396	***[I/V]***	partial

A small number of HLA-associated polymorphisms, including A*31:01-Gag-I401L and B*57:01-Nef-*x*133I, showed stronger population-level escape signal in early versus chronic infection (see Table 1). Though these differences were not statistically significant (Table 1, last two columns), we nevertheless hypothesized that they could represent potential examples of transient escape. In support of this hypothesis, the above table provides examples of two cases where a patient harbored the HLA-associated adapted variant at a given HIV-1 codon at the earliest timepoint post-infection, that subsequently give way to a non-adapted form (and/or a mixture of the two), consistent with transient early escape at these positions in these patients.

### Escape prevalence in early infection correlates with longitudinal first-year escape rates

**Figure 3:**
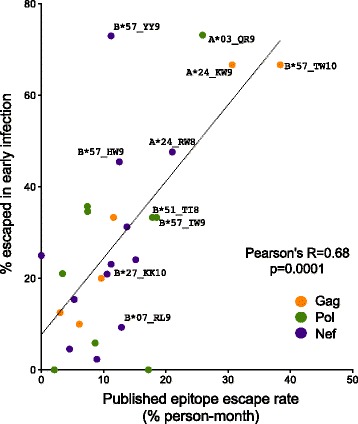
**Escape prevalence in early infection correlates with longitudinal first-year escape rates.** A total of 27 HLA-associated polymorphisms in Gag (orange), Pol (green) and Nef (purple) occurring within optimally defined CTL epitopes, for which first-year epitope-specific rates of escape were previously published [13], were investigated. A significant positive correlation is observed between the proportion of persons expressing the restricting HLA and harboring the relevant polymorphism (“proportion escaped”) in early infection and the published first-year epitope escape rate, providing proof-of-concept that the relative timecourse of early escape in HIV-1 can be inferred using cross-sectional methods. In the case where a given epitope contained multiple HLA-restricted polymorphic sites, the site exhibiting the maximum “proportion escaped” was used. For figure clarity, only a subset of well-known epitopes are labeled for interest.

Another objective was to investigate to what extent cross-sectional data could be used to infer the extent and time course of immune-driven HIV-1 adaptation. As such, we first wished to demonstrate that early escape frequencies calculated cross-sectionally predict rates of immune escape calculated longitudinally. Published first-year rates of escape were available for 27 optimally-described CD8+ T-cell epitopes [13] which contained one or more HLA-associated polymorphisms investigated in the present study. For example, the estimated first year escape rate for the Gag-TW10 epitope (Gag_240–249_) is 38.36% per person-month [13], while the prevalence of the Gag-T242N mutation among B*57-expressing persons in our early dataset is 67% (Figure 3). As expected, longitudinal first-year CD8+ epitope escape rates correlated significantly with HIV-1 polymorphism prevalence among persons expressing the relevant HLA in our early infection dataset (Pearson’s R = 0.68, p = 0.0001; Figure 3). Because ~40% of the patients in the present early infection cohort were included in the published longitudinal study [13], we re-analyzed our data with these overlapping patients removed, and observed that the correlation remained strong (Pearson’s R = 0.55, p = 0.0035, not shown). This supports HLA-associated escape mutation prevalence calculated cross-sectionally at < 3 months post-infection as a reliable surrogate marker of first year escape rates calculated longitudinally.

### Inferring the extent of host adaptation via comparative analysis of cross-sectional data from early and chronic infection

**Figure 4:**
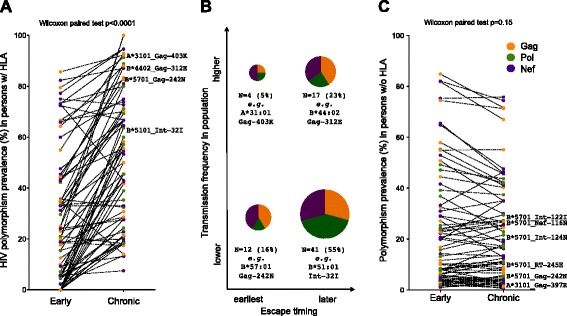
**Estimated extent of escape and reversion between early and chronic infection.** ***Panel A***: For each of the specific HLA-associated “adapted” (escaped form) HIV-1 polymorphisms investigated (N = 74 total), the proportion of persons expressing the restricting HLA and harboring the relevant polymorphism (“proportion escaped”) in early versus chronic infection are depicted as linked pairs. The data indicate that, on average, escape prevalence in persons expressing the restricting HLA allele more than doubles between early and chronic infection. ***Panel B***: The N = 74 HLA-associated “adapted” polymorphisms are broken down in terms of their relative timeline of escape (“earlier” vs. “later”, where the former is defined as population-level signal of p < 0.05 in early infection) as well as their relative prevalence/transmission frequency in the population (“lower” vs. “higher”, where the former is defined as <30%). The size of each pie reflects the proportion of polymorphisms in each category, while the “pie slices” denote the breakdown of polymorphisms by HIV-1 protein (orange, green and purple for Gag, Pol and Nef respectively). Summary statistics and examples of HLA-associated polymorphisms in each category are also provided. ***Panel C***: The proportion of persons harboring an HLA-associated adapted HIV-1 polymorphism in the *absence* of the restricting HLA allele in early versus chronic infection is shown. The data are generally consistent with slow reversion of many transmitted escape mutations [7,14,34]. In all panels, polymorphisms are colored by HIV-1 protein: Gag (orange), Pol (green) and Nef (purple); those mentioned in the text are labeled.

We next wished to use our cross-sectional early and chronic cohorts to quantify the extent of HLA-driven escape occurring between these two infection stages. For this analysis, we specifically defined “escape” as the specific adapted viral form associated with a given HLA allele at a given HIV-1 codon - for example, Gag 242 N is the B*57:01-associated adapted form at this position. This adapted list comprised N = 74 HLA-associated polymorphisms (25, 24, and 25 in Gag, Pol and Nef respectively) (Figure 2 and Additional file 1). We calculated the prevalence of each of these polymorphisms in persons expressing the relevant HLA allele in our early versus chronic cohorts, thus allowing us to estimate the extent of within-host HIV-1 adaptation between these two stages. Overall, the median “percentage escaped” (defined as the % of individuals expressing the relevant HLA and harboring the HIV-1 polymorphism of interest) was 23.8% [Interquartile range (IQR) 5.3-44.4%] in early infection versus 55.1% [IQR 28.4-73.0%] in chronic infection (p < 0.0001; Figure 4A). This indicates that, on average, escape prevalence in persons expressing the restricting HLA allele more than doubles between these infection stages. Breaking the analysis down by HIV-1 protein, the median early versus chronic escape prevalence was 23.5% [IQR 15.1-49.7%] vs. 55.6% [IQR 29.7-85.7%] in Gag, 11.3% [IQR 1.2-33.3%] vs. 50.5% [IQR 25.2-69.2%] in Pol, and 31.3% [7.5-63.0%] vs. 54.6% [21.4-73.3%] in Nef (all p ≤ 0.001, not shown). This is consistent with early escape occurring predominantly in Gag and Nef [13,14,19], while escape in Pol is generally slower but nevertheless approaches comparable levels by chronic infection.

Though summary statistics are informative, individual polymorphisms differ widely in their timing and extent of selection over the infection course. For this reason, details on polymorphism prevalence in persons expressing vs. not expressing the relevant HLA in early and chronic infection, along with their statistical measures of association, are provided in the Additional file 1. We highlight some examples here. First, for a substantial minority of polymorphisms (notably those in Table 1), escape is rapid, reproducible and largely complete within < 3 months post-infection. For example, 67% of B*57:01-expressing individuals already harbored Gag-242 N in early infection, a proportion that increased to 83% in the chronic phase (Figure 4A). Noting that Gag-242 N frequency was only 5.8% among persons lacking B*57:01 in early infection (Additional file 1), these results are consistent with escape having already occurred in over two-thirds of B*57-expressing persons by < 3 months post-infection [2,13], with an additional minority escaping somewhat later.

While the prevalence of Gag-242 N is low in the general population (5.8% among B*57:01-negative persons and ~1% among persons lacking an allele belonging to the B58 supertype), other polymorphisms are quite prevalent in circulation, but are nevertheless significantly enriched among HLA-expressing persons in early infection (Figure 4A). In this case, their high early prevalence is attributable to both frequent transmission and reproducible early escape. For example, both the A*31:01-associated Gag-403 K and C*03:04-associated Nef-85 L polymorphisms are observed at >40% prevalence in HIV-1 subtype B sequences, but their prevalence is ~55% and ~72% respectively among persons expressing the relevant HLA < 3 months post-infection (Odds Ratios 1.5 and 2.4 respectively, p < 0.01, Table 1 and Additional file 1). The observation that population-level approaches are capable of detecting strong escape signals despite high polymorphism background frequencies has previously been demonstrated in high-powered chronic infection cohorts [11]; the present study extends this to demonstrate such signals can also be detected very early in infection, in more modestly-powered datasets. Overall, if one uses the original criterion of early population-level statistical signal of p ≤ 0.01 to define HIV-1 sites that predominantly escape early, 15% (11 of 74) of adapted polymorphisms fall into this category; using a more liberal threshold of p < 0.05, this increases to 21.6% (16 of 74) (Figure 4B).

The remaining 78.4% (58 of 74) polymorphisms generally reproducibly escape later than 3 months following infection (Figure 4B). It is important to note that later escape can occur because CTL responses against these regions generally arise later during infection (*i.e.* there is no immune pressure on these epitopes in early infection), or because CTL responses arise relatively early, but escape does not reproducibly occur rapidly in a significant proportion of individuals expressing the relevant HLA allele. Among these later-escaping polymorphisms are those whose population background (transmission) frequencies are generally low, and those whose background frequencies are generally high. The B*51:01-associated Integrase-32I polymorphism at position 5 of the B*51-restricted LI9 epitope (Integrase_28–36_) provides an example of the former. In early infection, its frequency in B*51:01-expressing persons is 5%, not significantly different from background, but this rises to 64% by chronic infection (Figure 4A and Additional file 1). The LI9 epitope is known to be consistently targeted in B*51-expressing persons early after infection [30,31]*.* The observation that this epitope ultimately escapes via Int-32I in >60% of B*51:01-expressing persons suggests this epitope is under strong, sustained and reproducible CD8+ T-cell pressure by B*51 *in vivo,* where delayed escape is likely explained by a combination of mutational/fitness constraints and both intra-individual (“vertical”) and inter-individual (“horizontal”) CD8+ T-cell immunodominance hierarchies [19,32,33].

An example of a later-escaping polymorphism with high population background frequency is B*44:02-Gag-312E. Located at position 7 of the B*44:02-restricted AW11 epitope (Gag_306–317_), it represents the HIV-1 subtype B consensus residue at this codon. Its >60% frequency in both B*44:02 and non-B*44:02-expressing persons in early infection reflects its high transmission frequency, rather than early selection by B*44:02. Nevertheless, by chronic infection, 83% of B*44:02-expressing persons harbored Gag-312E, consistent with later escape (Figure 4A). A full categorization of HLA-associated “adapted” polymorphisms in terms of “earlier” vs. “later” escaping (defined as early p < 0.05 vs. p ≥ 0.05 respectively) and “lower” vs. “higher” background (estimated transmission) frequency (defined as <30% vs. p ≥ 30% respectively), is provided in the Additional file 1. A graphic depicting the proportion of HLA-associated polymorphisms in each of these categories, broken down by HIV protein, is provided in Figure 4B.

The extent of reversion of HLA-associated polymorphisms over time can be similarly estimated by calculating their prevalence in persons *lacking* the relevant HLA in the early versus chronic cohorts. The overall median percentage of individuals harboring a given polymorphism in the absence of the restricting HLA allele was comparable in early (13.7% [IQR 4.9-34.2%]) and chronic (14.9% [4.2-34.9%]) infection (p = 0.15; Figure 4C), consistent with slow reversion reported for many polymorphisms [7,14,21,34]. Note that inferred reversion frequencies merit cautious interpretation in cases where polymorphisms are selected by multiple alleles (*e.g.* the seemingly stable prevalence of Gag-147 L in A*25:01-negative individuals is likely due in part to its selection by B*13:02 and B*57:01 [11], associations that were not investigated in the present study). Nevertheless, results confirmed that HLA-B*57:01-Gag-T242N reverts between early and chronic infection (though RT-245E, Int-122I, Int-124N, or Nef-116N revert slowly or not at all, as reported previously [2,21,35]). The reversion analysis additionally revealed novel sites of potential interest. For example, the early escaping A*31:01-Gag 397R polymorphism (Table 1 and [18,19]) displayed evidence of reversion, suggesting that this mutation may have a high fitness cost.

### Host adaptation-related features distinguish protective and non-protective HLA class I alleles

Lastly, we wished to identify adaptation-related features that discriminate protective from non-protective HLA alleles, defined here as their published hazard ratios for progression to AIDS [HR-AIDS] in natural history studies [36]. Although the timecourse of viral escape is influenced by complex factors including epitope immunodominance hierarchies, strength of selection, mutational/fitness constraints and transmitted virus characteristics [17,19,34,37-40], we reasoned that HLA alleles that restrict polymorphisms that are already highly prevalent in early infection (due to rapid escape and/or frequent transmission) would be generally unfavorable for HIV-1 control. Thus, for all HLA alleles for which ≥2 adapted polymorphisms were investigated in the present study (N = 17 alleles total), we computed their mean “percentage escaped” in early infection. That is, we took the prevalence of each of these adapted polymorphisms in persons expressing the relevant HLA allele in our early cohort (displayed in Figure 4A), and, for each HLA allele, computed the mean of these values.

**Figure 5:**
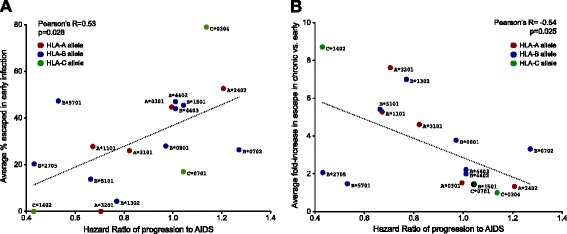
**Adaptation characteristics as correlates of HLA-associated progression risk.** Colored dots denote individual HLA-A (red), HLA-B (blue) and HLA-C (green) alleles for which ≥2 adapted polymorphisms were investigated in the present study (N = 17 alleles total). Each HLA allele’s Hazard Ratio of progression to AIDS (x-axis) was derived from historic published seroconverter studies [36]. ***Panel A***: For each HLA, the proportion of persons expressing that allele and harboring the specific viral HLA-associated polymorphism in early infection was calculated as the mean of all HLA-associated adapted polymorphisms investigated (y-axis). A significant positive relationship is observed between these two variables (Pearson’s R = 0.53, p = 0.028), suggesting that in general, high early escape prevalence is a correlate of higher HLA-associated progression risk. ***Panel B***: For each HLA, the mean fold-increase in escape in chronic versus early infection was calculated from all HLA-associated adapted polymorphisms investigated (y-axis). A significant inverse relationship is observed between these two variables (Pearson’s R = -0.54, p = 0.025), suggesting that in general, protective alleles are those from which HIV-1 escape is substantial and reproducible, yet occurs on a delayed timescale.

Consistent with our hypothesis, we observed a positive correlation between an HLA allele’s average extent of adaptation in early infection, and its HR-AIDS (Pearson’s R = 0.53, p = 0.028; Figure 5A). Of note, HLA-B*57:01 appears as somewhat of an outlier, exhibiting higher than expected escape prevalence in early infection given its protective nature. We hypothesize that the reason B*57 can maintain sustained HIV-1 control despite rapid escape in some epitopes (Table 1 and [2,13,16,18,28]) is because the early B*57-restricted CD8+ response often simultaneously targets more than one epitope, notably in p24^Gag^ [31,41-43], where escape is accompanied by fitness costs [44-46].

HLA-associated polymorphisms identified at the population level mark viral sites under strong, reproducible *in vivo* immune pressure by individual HLA alleles [11]. We thus further hypothesized that HLA alleles for which escape was substantial (*i.e.* selected in a high proportion of persons expressing the relevant HLA) but generally delayed (*i.e.* selected post early-phase) would tend to be more protective. As such, for the same set of HLA-associated polymorphisms we computed their fold-change in escape between early and chronic infection, and calculated the mean of these values per HLA allele. By this measure, alleles for which the majority of escape had already occurred in early infection would exhibit low subsequent fold-changes, whereas alleles selecting escape mutations that generally occurred later in infection would exhibit fold-changes reflecting the extent of selection pressure on these sites in persons expressing the relevant HLA. Consistent with our hypothesis, we observed an inverse correlation between an HLA allele’s HR-AIDS and its average fold-increase in escape in chronic versus early infection (Pearson’s R = −0.54, p = 0.025; Figure 5B). Of note, B*27 appears as an additional outlier in this analysis, possibly due to escape in the critical Gag-KK10 epitope requiring nearly a decade in some individuals [32,47,48] due to its high fitness/mutational barrier [33,49].

Overall, our findings are consistent with a high early burden of adaptation to host HLA (either via rapid escape or frequent polymorphism transmission) as a correlate of HLA-associated progression risk. Conversely, HLA alleles from which HIV-1 escape is substantial and reproducible yet occurs on a delayed timescale appears to be a correlate of protection. Taken together with observations that protective HLA alleles contribute substantially to the total HIV-specific CD8+ response in early infection [31,38], that they impose broad selection pressures on HIV-1 [11]; and that some CD8+ epitopes escape slowly despite sustained CD8+ targeting [19,48], results suggest that the capacity to exert consistent, substantial and sustained pressure, ideally on multiple epitopes, from which the virus can only escape on a relatively delayed timescale, is a correlate of protection.

Some limitations of our study merit mention. Firstly, our early and chronic datasets are relatively modestly powered by association testing standards, so it was not possible to examine all published HLA-associated polymorphisms in HIV-1 subtype B. Secondly, due to the lack of information on duration of infection for chronic patients, it is likely that the chronic cohort comprised patients at a range of infection stages. Inclusion of some chronic patients with less advanced infection could underestimate the extent of escape at this stage. Finally, although care was taken to match our early and chronic datasets as closely as possible for HIV and HLA genetic diversity and distribution, it is essentially impossible to achieve perfectly matched datasets. As such, we cannot rule out small differences in statistical power between cohorts, and therefore should interpret candidate “transient” early escape results with some caution.

## Conclusions

In conclusion, our results provide proof-of-concept that statistical association approaches can be applied to cross-sectional host/viral genetic datasets to identify the most rapidly selected HLA-associated polymorphisms in HIV-1 *that are also reproducibly selected across patients*. As such, results from these types of population-level studies complement those of individual-level longitudinal analyses that cannot assess inter-patient reproducibility. Furthermore, the extent and relative timing of individual escape events (in terms of early versus later in the infection course) can also be inferred from cross-sectional data. In particular, we demonstrate that high escape prevalence in early infection (either due to rapid selection and/or high transmission frequency) is a correlate of HLA-associated progression risk while reproducible later escape (a surrogate of consistent immune selection on a given site in persons expressing the relevant HLA) is a correlate of protection.

Given that longitudinal observational studies of untreated persons are incompatible with current recommendations for early HIV-1 treatment initiation [20] and treatment as prevention [50], cross-sectional analyses of pretreatment host/viral genotypes could provide relevant alternative tools to advance our knowledge of HIV-1 adaptation, including the earliest events post-infection. We suggest that studies such as the present one be undertaken with expanded cross-sectional cohorts, comprised of individuals at various clinical stages of infection, including from different HIV-1 subtypes.

## Methods

### Early and chronic infection cohorts

The early cohort was comprised of HIV-1 subtype B infected patients recruited through various observational seroconverter studies including the Acute Infection and Early Disease Research Program (AIEDRP) sites in Boston and New York (USA), Sydney (Australia), a private medical clinic in Berlin (Germany), and observational cohort studies in Montreal and Vancouver (Canada) [13,23,24]. Infection dates for the patients in the early cohort were estimated as described in [13,23]. Briefly, for patients with positive HIV RNA (>5,000 copies/ml) or detectable serum p24 antigen but a negative HIV-1 enzyme immunoassay (EIA), 4 weeks were subtracted from the negative EIA date. For patients with positive EIA but negative/indeterminate Western blot, 6 weeks were subtracted from the positive EIA date. For patients with negative detuned EIA, 4 months were subtracted from this date. For the remainder, infection dates were estimated as the midpoint between the last negative and the first positive HIV test. Clinical histories were incorporated into infection date estimates where available.

To maximize our power to detect HLA-associated polymorphisms in the early infection stage, all available early infection patients were included in the present study. For each of these patients, the sample closest to ~3-months following the estimated date of infection was selected, yielding a median sampling distribution of 88 days [Interquartile Range 63–120 days] post-infection for early samples. In contrast, the chronic cohort was assembled from the baseline (pre-therapy) timepoint from a total of more than 300 HIV-1 subtype B infected individuals initiating antiretroviral therapy in British Columbia, Canada, and untreated HIV-1 subtype B infected individuals in Boston, USA [13,25]. Time since infection is unknown for individuals in the chronic cohort, however the median CD4 count at sampling was 250 [IQR 147–360] cells/mm^3^ for this group. To create HIV-1 gene-specific chronic infection datasets of equal size to the early cohort, that were also matched as closely as possible for HLA class I and HIV-1 diversity of the early cohort, chronic patients were selected from the total group using an iterative process to achieve the closest matching of HIV-1 and HLA distributions (Figure 1). All early and >75% of chronic patients were antiretroviral naïve; the remainder were untreated at time of sampling.

#### Ethics statement

All patients provided written informed consent. Ethical approval was obtained through the institutional review boards at the Massachusetts General Hospital, the BC Centre for Excellence in HIV/AIDS and Simon Fraser University.

### HIV-1 and host (HLA class I) genotyping

HIV-1 RNA was extracted from plasma using standard methods. Gag, Pol (including protease, codons 1–400 of Reverse Transcriptase, and Integrase), and Nef were amplified in separate nested RT-PCR reactions using HIV-1 subtype B-specific primers. Amplicons were bulk-sequenced bidirectionally on a 3130xl and/or 3730xl automated DNA sequencer (Applied Biosystems). Chromatograms were analyzed using Sequencher v5.0 (Genecodes) or RECall [51] with nucleotide mixtures called if the height of the secondary peak exceeded 25% of the height of the dominant peak (Sequencher) or 20% of the dominant peak area (RECall). HIV-1 sequences were confirmed as subtype B using the recombinant identification program (RIP; http://www.hiv.lanl.gov/content/sequence/RIP/RIP.html) and aligned to the HIV-1 subtype B reference strain HXB2. Phylogenetic trees were constructed using PhyML [52] and visualized using FigTree (http://tree.bio.ed.ac.uk/software/figtree/). Pairwise genetic distances were computed from newick treefiles using PATRISTIC [53]. HLA class I typing was performed using sequence-based methods [54] and imputed where necessary to high resolution using a machine learning algorithm ([55]; http://research.microsoft.com/en-us/projects/bio/mbt.aspx#HLA-Completion). HIV-1 sequences from cohorts where REBs allow public sequence deposition have been deposited in GenBank: accession numbers are Gag (KJ869442 - KJ869609), Protease-RT (KJ869900 - KJ870015), Integrase (KJ869610 - KJ869735), Nef (KJ869736 - KJ869899). A full summary of polymorphism frequencies, broken down by HLA allele carriage and infection stage is provided as Additional file 1.

### Definition and identification of HLA-associated polymorphisms

The published reference list of N = 453 HLA-associated polymorphisms in HIV-1 subtype B Gag, Pol and Nef sequences was defined in an independent international cohort of >1800 individuals chronically infected with HIV-1 subtype-B using phylogenetically-informed methods at q < 0.05 [11]. The cohort used to define these associations had no overlap with the early and chronic cohorts studied here [11]. Briefly, to identify HLA-associated polymorphisms in linked HIV/HLA datasets, maximum likelihood phylogenetic trees (one per HIV-1 gene) are constructed, and a model of conditional adaptation is inferred for each observed HIV-1 amino acid at each codon. The amino acid is assumed to evolve independently along the tree until it reaches the tips, representing the present host. Selection via host HLA-mediated pressures and HIV-1 amino acid covariation is directly modeled using a weighted logistic regression, in which the individual’s HLA repertoire and covarying HIV-1 amino acids are used as predictors, and the bias is determined by the inferred possible transmitted sequences (as inferred via reconstruction of the amino acid frequencies at the penultimate internal nodes in the phylogeny) [12]. Here, the null hypothesis is that the observed amino acids at the tree tips are explained by the phylogeny only, whereas the alternative hypothesis is that they are better explained by the presence of a specific HLA (or covarying HIV-1 amino acid) in the present host. To identify which factors (HLA and/or HIV-1 covariation) contribute to the selection pressure, a forward selection procedure is employed where the most significant association is added to the model in an iterative fashion, with p-values computed using the likelihood ratio test. Statistical significance is reported using q-values [56], the p-value analogue of the false discovery rate (FDR). Q-values denote the expected proportion of false positives among results deemed significant at a given p-value threshold; for example, at q ≤ 0.05, we expect 5% of identified associations to be false positives.

HLA-associated polymorphisms are classified into two categories: (1) “Adapted forms”, amino acids significantly *overrepresented* in the presence of the HLA allele in question, which represent the putative escape forms associated with that HLA at that codon, and (2) “Nonadapted forms”, amino acids significantly *underrepresented* in the presence of the HLA allele in question, which represent the immunologically susceptible form associated with that HLA at that codon. In most cases, HLA-associated nonadapted forms represent the subtype consensus amino acid while adapted forms represent polymorphic variants – but exceptions exist.

To identify an appropriate subset of known HLA-associated HIV-1 polymorphisms that are appropriate for study in datasets of the present size (N ~ 200) and host/viral genetic distribution, we interrogated our early and chronic infection cohorts for these N = 453 published polymorphisms [11] using the phylogenetically-corrected methods described above. As described in the results, this yielded a subset of N = 162 HLA-associated polymorphisms detectable in our early and/or chronic datasets (Figure 2 and Additional file 1).

Our analyses also featured comparisons of the strength of selection of HLA-associated polymorphisms between early and chronic cohorts, undertaken using a previously-described phylogenetically-corrected interaction test [12,22,27]. Briefly, we took the union of all HLA-associated polymorphisms detectable at the population level in either the early or chronic cohorts (N = 162). For each association on the list, we constructed a phylogenetically-corrected logistic regression model using the restricting HLA as a predictor. Using a likelihood ratio test, we compare this model to a more expressive one that includes an additional interaction term that assigns “1” if the individual expresses the restricting HLA allele and is in the chronic cohort, or “0” otherwise. This allows us to obtain a p-value testing the null hypothesis that HLA-associated selection at that site is not significantly different in early versus chronic cohorts.

#### Statistical analyses

Fisher’s exact test was used to compare HLA class I allele frequencies between cohorts. The Mann–Whitney paired test was used to compare the prevalence of HLA-associated polymorphisms in the presence/absence of their restricting HLA, in early versus chronic cohorts, as these data were non-normally distributed. Pearson’s correlation was used to investigate the relationship between early escape prevalence and published first-year rates of escape [13], as well as with published HLA allele-specific Hazard Ratios for progression to AIDS [36], as these data did not significantly violate the assumption that values were drawn from a normal distribution. In single analyses, significance is denoted by p < 0.05. In the case of multiple tests, q-values are used [56]; thresholds are defined throughout the paper. All tests of significance were two-tailed.

## Additional file

Additional file 1:
**A full summary of HLA-associated polymorphisms, their HIV genomic locations and directions of association (adapted vs nonadapted), and their observed frequencies in early versus chronic infection in persons harboring vs. not harboring the restricting HLA allele are provided in columns A-L and O-T.** P-values and q-values of association are also provided for early infection (Columns M-N) and chronic infection (Columns U-V). P- and q-values comparing the strength of selection of individual HLA-associated polymorphisms between early and chronic stages are provided in Columns W-X. Finally, all HLA-associated “adapted” polymorphisms are categorized with respect to their relative timescale of escape (early vs. later; Column Y) as well as their relative background frequency in the population (lower vs. higher; Column Z).
